# Machine learning revealed symbolism, emotionality, and imaginativeness as primary predictors of creativity evaluations of western art paintings

**DOI:** 10.1038/s41598-023-39865-1

**Published:** 2023-08-10

**Authors:** Blanca T. M. Spee, Jan Mikuni, Helmut Leder, Frank Scharnowski, Matthew Pelowski, David Steyrl

**Affiliations:** 1 Vienna Cognitive Science Hub, University of Vienna, 1010 Vienna, Austria; 2 Donders Institute for Brain, Cognition and Behavior, Department of Neurology, Center of Expertise for Parkinson & Movement Disorders, Radboud University Medical Center, 6525 GC Nijmegen, The Netherlands; 3 Department of Cognition, Emotion, and Methods in Psychology, Faculty of Psychology, University of Vienna, 1010 Vienna, Austria; 4 Department of Psychiatry, Psychotherapy and Psychosomatics, Psychiatric University Hospital, University of Zurich, 8008 Zurich, Switzerland

**Keywords:** Psychology, Human behaviour

## Abstract

Creativity is a compelling yet elusive phenomenon, especially when manifested in visual art, where its evaluation is often a subjective and complex process. Understanding how individuals judge creativity in visual art is a particularly intriguing question. Conventional linear approaches often fail to capture the intricate nature of human behavior underlying such judgments. Therefore, in this study, we employed interpretable machine learning to probe complex associations between 17 subjective art-attributes and creativity judgments across a diverse range of artworks. A cohort of 78 non-art expert participants assessed 54 artworks varying in styles and motifs. The applied Random Forests regressor models accounted for 30% of the variability in creativity judgments given our set of art-attributes. Our analyses revealed symbolism, emotionality, and imaginativeness as the primary attributes influencing creativity judgments. Abstractness, valence, and complexity also had an impact, albeit to a lesser degree. Notably, we observed non-linearity in the relationship between art-attribute scores and creativity judgments, indicating that changes in art-attributes did not consistently correspond to changes in creativity judgments. Employing statistical learning, this investigation presents the first attribute-integrating quantitative model of factors that contribute to creativity judgments in visual art among novice raters. Our research represents a significant stride forward building the groundwork for first causal models for future investigations in art and creativity research and offering implications for diverse practical applications. Beyond enhancing comprehension of the intricate interplay and specificity of attributes used in evaluating creativity, this work introduces machine learning as an innovative approach in the field of subjective judgment.

## Introduction

Creativity can be found in many domains and within various human endeavors, ranging from art and science to social gaming and creativity in daily life^1–3^. Almost indisputably, creativity is strongly associated with the fine arts, particularly visual arts^4^. This association is not only valid under consideration of sociological aspects^5^ but is also scientifically substantiated. Representative here is the collection of 225 creativity tests by Torrance and Goff^6^, where a significant portion of tests fall into the category of “figural image-making”. Creativity is also esteemed as an estimation marker for often pragmatic ulterior motives, such as the selection of artworks made by museum curators to attract museum visitors^7^, scoring individuals on creativity as a parameter to attend art schools, and the assessment of artworks in terms of their aesthetic and qualitative value^4,8^. In addition—and looking at what impact creative engagements may have on people—creative art interactions are considered pathways to exploration, problem-solving^9,10^, and communication channels for individuals and societal discourse^5^. Thus, artworks are valued as creative icons not only for their beauty or elegance, but paintings, drawings, and even scribbles from famous artists—like the one-line animal sketches by Pablo Picasso—are also appreciated because they are judged as creative.

Artworks as creative products in all their diversity have become ubiquitous in human societies and serve as central commodities for inter-social and cultural comparisons and benchmarks^5,11–13^. The judgment of creativity plays hereby a pivotal role in establishing such benchmarks^14^, where the degree of creativity determines the status of artists or geniuses, the famousness of an artwork^15^, its monetary value and the degree of copyright protections^16^. The relevance of creativity becomes even more evident when considering its consistent presence in the discourse surrounding the conceptualization of art across cultures and historical periods, where creativity appears to be universally applied as an art judgment for all kinds of visual art forms, innovations, and at different historical time-points^3^.

However, despite its fundamental importance in artistic and societal domains, creativity remains a polysemous concept lacking a clear definition, even in terms of its meaning as an art judgment^17^. Consequently, due to its multifaceted nature, researchers have employed various approaches, both objective and subjective, to measure creativity. On the one hand, studies have focused on objective formal-perceptual and design-oriented features, such as the Golden Ratio and luminance, comparing them to subjective preference ratings using classical and statistical learning methods^18–21^. Computational aesthetics has also evaluated aesthetic measures and quantification to generate designs (e.g.^22,23^). However, to the best of our knowledge, none of these studies, also the ones using statistical learning methods for objective measures, have centrally investigated creativity as a judgment and examined subjectively perceived attribute structures along this art evaluation.

On the other hand, researchers in visual arts, creativity, and psychology typically measure creativity levels through subjective assessments of the final products using mainly linear statistical methods (^3^, see for review^24,25^). Such assessments have been employed in art viewing studies, including, e.g., standardized tests where participants complete drawings and provide subsequent scores or ratings^26^. These assessments gained prominence following the seminal work of Daniel Berlyne^27–29^, a central figure in art research who primarily studied art-specific properties through preference judgments. Subsequent studies have focused on composition comparisons^30^, evaluation of realistic copying^25^, differences between trained artists and amateurs, and distinctions in technique, styles, artists’ traits, states, and skill sets. For example, Hager et al.^31^ included creativity in their art reception survey connecting through principal component analysis artistic quality and creativity with five personality factors encompassing cognitive engagement, comprehension, affective appraisal, self-referential, and knowledge aspects. Hence, creativity judgments in art and within other creative domains are often centered around studying exceptionally creative people along their accomplishments^32^. Another example is the Consensual Assessment Technique (CAT)^30,33,34^, which involves participants sorting creative products into groups based on their perceived levels of creativity. Notably, studies applying the CAT, also to Picasso’s work^35^, have found high coherence even among non-art experts (see also Amabile^30^ reporting a high inter-rater-reliability ranging from 0.72 to 0.93 in 20 studies in the visual art domain).

In all these studies, creativity judgments rely on people’s ability and coherence to recognize and judge what is creative or not and to explain their decision along attributes^30,31,34^. This interactive use of terminology to communicate opinions and justification using attributes appears to be a result of social-cultural and educational training in interacting with aesthetic goods^36^, and certainly might differ depending on social-cultural background and expertise^37,38^. Similarly, in scientific psychological assessments, there is a common practice of establishing correspondence between the concept of a term and behavioral outcomes^3,4^. Hence, studying the complex associations between judgment and attributes that can potentially be incorporated into numeric predicting patterns^4,5,36,39^, is intriguing considering that individuals appear to be adept at recognizing creativity.

However, previous studies have not addressed the underlying question of how individuals identify creative art based on specific attributes. Neither of the studies used subjectively perceived content-representational and formal-perceptual attributes predicting creativity judgements (see “Materials and methods” for attribute list). In addition, most studies used statistical models assuming linear associations between independent and dependent variable, i.e., changes in art-attribute scores would correspond directly to changes in creativity judgments with a constant factor. Human behavior, however, is most likely not based on such linear associations and such a simplification can lead potentially to irrelevant theories and questionable scientific conclusions^40^. Exemplary here is, again, Berlyne’s and later research findings indicating that preferences, such as liking or appreciation, are not linearly associated with attributes like complexity; instead, at a specific threshold, judgments exhibit a sudden shift, leading to a pronounced increase or decrease in liking^29,41–44^. This phenomenon, which might also extend to the realm of creativity, suggests the association of unknown attributes influencing the perception and evaluation of creative works might also not be linear. These issues led us to the following research question: Which subjectively perceived art-attributes contribute to the judgment of an artwork’s level of creativity?

To address this gap, we conducted a study using 17 visual art-attributes assessed through semantic differential scales^45^. These attributes, such as warm/cold, simple/complex, emotionless/emotionally loaded^46^, have been identified in art research as influential factors in rating artworks^14,28,47–50^. We employed Random Forest machine learning regression models, which can learn non-linear association patterns and interactions from data^39^, to predict creativity judgments based on the aforementioned attributes. To analyze the importance of each individual art-attribute in predicting creativity judgments, we utilized permutation importance, a method from the interpretable machine learning field^6^. Our hypothesis is that art-attributes enable the prediction of creativity judgments, and our analysis of the model will identify art-attributes that contribute significantly to the prediction. By adopting this exploratory approach that encompasses multiple attributes, we broaden the scope of statistical designs typically employed in art research, opening new avenues to delve into the intricacies of judgment behavior, particularly within the domain of creativity in visual art. Furthermore, our study presents a quantitative and testable model that elucidates the statistical interplay between art-attributes and assessments of creativity. As a result, our findings have implications not only for practical applications but also for socio-cultural understanding across a diverse range of scientific fields. Moreover, this methodological framework can be adapted for investigating other types of judgments and participant samples, further expanding its utility and applicability.

## Materials and methods

### Sample size

Precise sample size justification (power analysis) for complex machine learning-based data analysis methods is still an open matter, and to the best of our knowledge, no standards have been established. Therefore, we followed a series of available suggestions regarding a reasonable sample size. First, a commonly disputed suggestion is that 50 samples are required to start any meaningful machine learning-based data analysis (scikit-learn 2021^51^). Second, a controversial suggestion is that 10 to 20 samples per degree of freedom (independent variable, art-attribute) is reasonable, particularly for logistic regression, which would result in a total of 170 to 340 samples needed for our study^52^. Third, a traditional power analysis using the two-tailed Student’s t-test, with an alpha of 0.05 and a power of 0.8, suggests that 394 samples would be required to detect small effects (Cohen’s d 0.2^53^). Generally, a larger sample size allows for the detection of smaller effects, as is the case with other statistical data analysis methods. Based on these considerations and available resources, we collected a total of 4206 samples (78 participants × 54 images, with 6 ratings missing due to recording issues). This number adheres to best practices in artwork rating studies^26,54^.

### Participants

Ratings were made by a final sample of 78 psychology students recruited at the University of Vienna (55 female, *M*_*age*_ = 24.23, *SD* = 3.45, ranged from 19 to 35). All participants signed an informed consent. All participants were fluent in German (surveyed via mother tongue), were informed of the purpose of the study, and participated for course credits. Raters were all assessed in terms of their self-reported art expert level (*M* = 2.23, *SD* = 1.27), their art education on art theory (*M* = 1.14, *SD* = 0.59), on art history (*M* = 1.13, *SD* = 0.55), on studio art (*M* = 1.13, *SD* = 0.48), their self-reported art-making expertise (*M* = 1.27, *SD* = 0.47) with 7-point Likert visual scales (1: does not at all apply to me, 7: completely applies to me), and answered the Vienna Art Interest and Art Knowledge (VAIAK) Questionnaire to assess their art interest and knowledge^55^. The art interest scores ranged from 12 to 71 across participants; the mean art interest score across all participants was 40.00 (*SD* = 13.90). Note that the possible range of the art interest score is from 7 to 71. The art knowledge scores ranged from 2 to 20 across participants; the mean art knowledge score across all participants was 7.03 (*SD* = 3.75). Note also that the possible range of the art knowledge score is from 0 to 36. Given all ratings and scores from our sample, it is plausible to assume that they can be identified as general art novices (see^55^ for rationale). This study was conducted in accordance with the guidelines and regulations outlined by the Ethics Committee of the University of Vienna (reference number ethics committee 00256), which approved the research involving human participants. Informed consent was obtained from all participants, all methods were performed in accordance with the relevant guidelines and regulations approved by the Ethics Committee, adhering to the principles set forth in the Declaration of Helsinki.

### Procedure

The testing was conducted in a laboratory room with four separate workstations, and participants were positioned approximately 50 cm from the screen. Participants received written instructions stating that they would be shown a series of visual artworks and were asked to provide ratings for each image using a set of attribute items, including the creativity judgment. It is important to note that in this paper, we focused our analysis solely on the creativity judgments along the attributes. Participants were allowed to take as much time as they needed to complete the ratings and could take short breaks if necessary. The artworks were displayed on a 19ʹʹ Iiyama ProLite B1906S monitor, with the longest dimension of each artwork fixed at a maximum of 500 pixels (1280 × 1024, 60 Hz resolution).

To evaluate the artworks, the images were shown individually and centered on the screen, with a top margin distance of 1 inch and a white background. Below each image, the attribute items and judgments were presented, and participants could scroll down using the mouse while keeping the artwork image fixed on the screen. Each artwork was presented once to each participant. Each participant rated the full set of artworks, resulting in a total of 4206 ratings (including 17 art-attributes and creativity judgments, with 6 ratings missing due to recording issues). The order of the rating items and artworks was randomized between participants, and the rating items were randomized for each image presentation (per trial) to avoid rating sequence effects.

The set of art-attribute items was presented as bipolar scales (semantic differentials), with a slider positioned in the middle between the two poles on a 100-point Likert scale. The full list of items for the attributes, along with their dimensions and the exact wording in German, is provided in Supplementary Information Table S1, and the English version is described in the following subsection (also see Table 1).

**Table 1:** Items of art-attributes along their semantic differential dimension poles (independent variables used in machine learning analysis).

Attributes	Instructions	Please evaluate the artwork based on the different attributes	
	Items	Negative pole (minimum)	Positive pole (maximum)
i. Formal-perceptual attributes	a. Visual harmony (balanced)	Visual harmony, proportional	Peculiar, strange shapes
	b. Depth	Two-dimensional	Three-dimensional
	c. Complexity	Simple	Complex
	d. Color saturation	Soft, pastel	Intense, strong
	e. Color variety	Few colors	Many colors
	f. Color temperature	Warm colors	Cold colors
	g. Color world	Dark color world	Light color world
	h. Brushwork	Fine brushwork	Rough brushwork
	i. Utilization of drawing area	Little utilization of the painting area	Full utilization of the painting area
ii. Content-representational attributes	j. Abstraction	Representative	Abstract
	k. Imaginativeness	Realistic content/topic	Imaginary, unrealistic, fantastic
	l. Symbolism (ambiguity)	Distinct/clear (unambiguous interpretation)	Symbolic (more space for interpretation, ambiguous)
	m. Accurate object representation	Photorealistic	Painterly
	n. Liveliness/animation	Dynamic	Still
	o. Emotionality	emotionless	Emotionally loaded
	p. Valence	negative valence	Positive valence
	q. Focus	Much context/environment in the image	Focused content

English version (for German version see Table S1 in Supplementary Information).

Note, that we decided not to cover attributes of eliciting emotional experience triggered in the person (for example, “the artwork makes me sad, happy, or aroused”). We made this decision to preserve a subjectively evaluated art-attribute specific focus consistent with many of the ratings used in earlier art research literature^28,41,64^ ﻿and not to conflate it with emotional self-focused consequences, which has resulted in diffuse scale groups in many of the previous studies (^63^, see e.g.^65^). We also had to decide to limit the items due to time expenditure and influence of fatigue. Lastly, we chose attributes that might also be used for other creative expressions, traditions, and cultures. Furthermore, the attributes could also be understood as observer responses using attributes to justify judgment (as discussed in the Introduction). However, we base our research on earlier art research using attributes for evaluating visual art and therefore follow that tradition in evaluating judgment behavior in that manner.

At the end of the testing session, all participants answered questions about their art expertise, education, and experience in making art. Additionally, they responded to the questions included in VAIAK^55^ to measure their art interest and knowledge scores.

### Art-attribute selection for rater assessments

Our focus was on the judgment of an artwork’s *creativity,* by asking: “How creative do you find the artwork?”. To evaluate the independent variables for this judgment we used art-attributes representing *formal perceptual* and *content-representational* dimension poles. We based our art-attribute set on previous literature, where the Assessment of Art Attributes battery (“AAA”^56^) presented a most suitable and well-educated set of attributes.

The AAA covers six formal-perceptual attributes (balance, color saturation, color temperature, depth, complexity, brushstroke) and six conceptual-representational attributes (abstractness, vividness, emotionality, imaginativeness, factual accuracy, symbolism), which we also used, in a slightly adapted version, in our study. We adapted the scales suitable for German terminology usage. We also used coherent semantic differentials (word-pair pole) introduced by Osgood et al.^57^, an approach commonly applied in many research fields^58^.

We further added the following art-attributes: in the color sector for completeness we added *color variety* with the opposite poles few and many colors and *color world* with dark/light color as opposite poles^59–61^; we added utilization of drawing area (little/full utilization) and focus (much context/focused content) as two attributes respecting different cultural background (^60^, e.g., Asian vs. European foci^62^); last, we included valence as one of the most commonly used art-attributes in empirical art research^8,54^. Our art-attributes including opposite attributes pole dimensions are listed in Table 1 (see for German version Supplementary Information Table S1).

### Stimuli

The stimuli were a selection of 54 images (2D static images of visual artworks) chosen from the Vienna Art Picture System (VAPS), which is a dataset of 999 fine art paintings and subjective ratings for art research^66^. The artworks differ in the depicted motif (genre categories) and stylistic characteristics (style, historical period subcategories) and were developed to be applicable for research purposes assuring high-quality images. All artworks are fine art originated in a Western culture. We used three genres (1) portrait, (2) landscape, and (3) still life, and three style-categorization: (1) representative (Baroque and Rococo images), (2) impressionistic (Impressionsism and Post Impressionism), and (3) abstract art (Cubism and Surrealism; for full list of artworks Table S2 see Supplementary Information; see for pre-ratings and stimulus selection Supplementary Information text and Table S3). We showed the same number of images in terms of depicted motif and style.

### Machine learning based data analysis approach

The machine learning-based regression analysis was performed in Python v3.8.5 using scikit-learn library v0.23.2^51^. In the analysis, the independent variables were visual harmony (balanced), depth, complexity, color saturation, color variety, color temperature, color world, brushwork, utilization of drawing area, abstraction, imaginativeness, symbolism (ambiguity), accurate object representation, liveliness/animation, emotionality, valence, and focus, whereas the creative judgment was the dependent variable.

Random Forests (RF) ensemble models were chosen to perform the actual regression task because this machine-learning method is computationally efficient and highly accurate^39^. It can inherently model associations between independent and dependent variables that go beyond a constant factor (that is non-linear associations) as well as variable interactions^39^. Furthermore, RF ensemble models are robust against multicollinearity in data^39^. Regression performance—the prediction accuracy—was assessed using a nested cross-validation (CV) procedure^67^. CV implements repeated train-test splits of the data to assess the generalizability of a model to new unseen data. A shuffle-split scheme with 128 repetitions (20% of the participants in the testing-set, 80% of the participants in the training-set) was applied in the main (outer) CV loop. In each repetition, the training-set was used for data scaling (z-scoring) and model complexity optimization. Model complexity optimization was carried out in a nested (inner) CV procedure using a sequential Bayesian optimization procedure in combination with again a shuffle-split scheme (20% testing, 80% training, 128 repetitions, 96 initial points) to find the best-performing complexity parameters (BayesSearchCV, scikit-optimize, v0.8.1, min_samples_split 2 to 128, min_samples_leaf 1 to 128, max_features 1 to total number of features). The complexity parameters that led to the highest prediction accuracy in the training-set of the inner CV were subsequently used along with the constant parameters n_estimators = 256, criterion = friedman_mse and all other parameters left to default, to train RF regressor models in the main CV loop using the training dataset only. The models were subsequently tested on the respective testing-set of the main CV loop. The testing-set was explicitly not used in the inner CV loop. Regression performance was measured with (1) the prediction coefficient of determination (prediction *R*^2^), and (2) the mean absolute error^68^. *R*^2^ scores are scaled that 0 equals a performance as good as using the average value of the dependent variable as predictor (the trivial predictor), and that 1 means no error at all. However, *R*^2^ scores can have values between minus infinity (a model that performs worse than using the mean value of the dependent variable as prediction) and 1 (perfect model, no error at all). Notably, the prediction *R*^2^ will be smaller than *R*^2^ values of conventional statistical models because the prediction *R*^2^ measures prediction performance for unknown data and not post hoc model fit. The mean absolute error reflects the average error that is made at each prediction and is not normalized; hence, it is measured in the scale of the dependent variable, the creativity rating (1–101).

The importance of single independent variables for the prediction (regression performance) was assessed with a permutation procedure^46^. For that, the change in regression performance was measured between original data and when single independent variables values were permuted between data instances. The drop in regression performance (drop in prediction *R*^2^) is a direct measure for the importance of the permuted independent variable^46^.

Statistical significance of the prediction *R*^*2*^ metric and of the independent variables’ importance were assessed using a shuffle (randomization) testing procedure a.k.a. exact test^71,72^. After evaluating the regression performance, the model was refitted using the same training data, but with shuffled dependent variable values. The shuffling was repeated 64 times in each main CV loop—16,384 times in total—to assess how likely it is that the obtained prediction *R*^2^ (original data) and the associated independent variable importance’s appear by chance^71,72^. The Null hypothesis was that there is no association between independent and dependent variables, and therefore, the obtained *R*^2^ and independent variable’s importance’s appear by chance. To reject the Null hypothesis at 95% confidence, it is not allowed that more than 5% of the metrics obtained with shuffle data are more extreme than the metric obtained by the original data^71,72^.

The influence of independent variables on the prediction can vary across the range of these variables due to the capacity of RF models to capture non-linear associations between independent and dependent variables. Therefore, the strength of these associations can exhibit variability across different levels of the independent variables. Partial dependence analysis was applied to assess these non-linear associations^46^. Partial dependence analysis computes the expected (average) model prediction for an independent variable’s range.

## Results

We predicted creativity judgement ratings of artworks based on ratings of 17 visual art-attributes using machine learning. We computed multivariate Random Forest (RF) regressor models and determined their prediction performances. On average the RF model’s predicting creativity judgements were off by 17.5 points from the actual judgements, within a creativity judgement range of 1 to 101 (see Table 2). The coefficient of determination (*R*^2^) between the predicted creativity judgements and the actual creativity judgements was on average 0.30, which indicates that the RF models explain on average 30% of the total variance in creativity judgement ratings.

**Table 2:** Performance of Random Forests regressor predicting creativity ratings.

Scales of art-judgments	Average mean absolute error (MAE) ± standard deviation	*p* value of bigger or equal than shuffle data MAE	Average coefficient of determination (*R*^2^) ± standard deviation	*p* value of smaller or equal than shuffle data *R*^2^
Creativity	17.5 ± 0.94	*p* < 0.001	0.30 ± 0.05	*p* < 0.001

Average and standard deviation from 128 repetitions of cross-validation.

**Figure 1:**
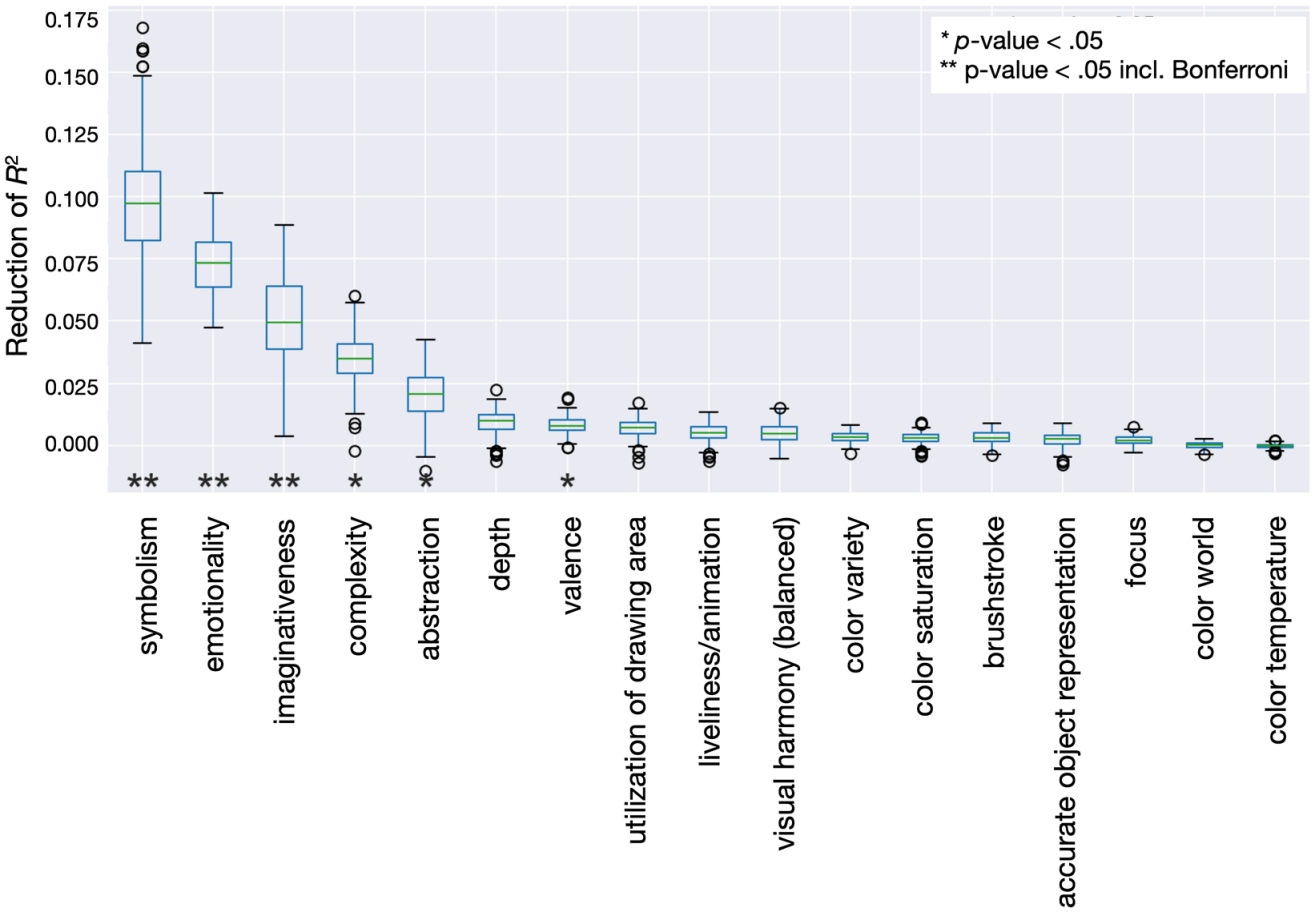
Art-attributes importance’s for predicting creativity. Art-attributes importance’s were assessed by a permutation procedure. The reduction in *R*^2^ after shuffling values of a specific art-attribute represents a direct measure of their importance for the prediction of creativity ratings.

We analyzed the contributions of single visual art-attributes (independent variables) to the RF model prediction performance, indicating which visual art-attributes are important to predict the creativity judgment (see Fig. 1). We used a permutation-based variable importance measure and shuffle (randomization) significance tests.

**Figure 2:**
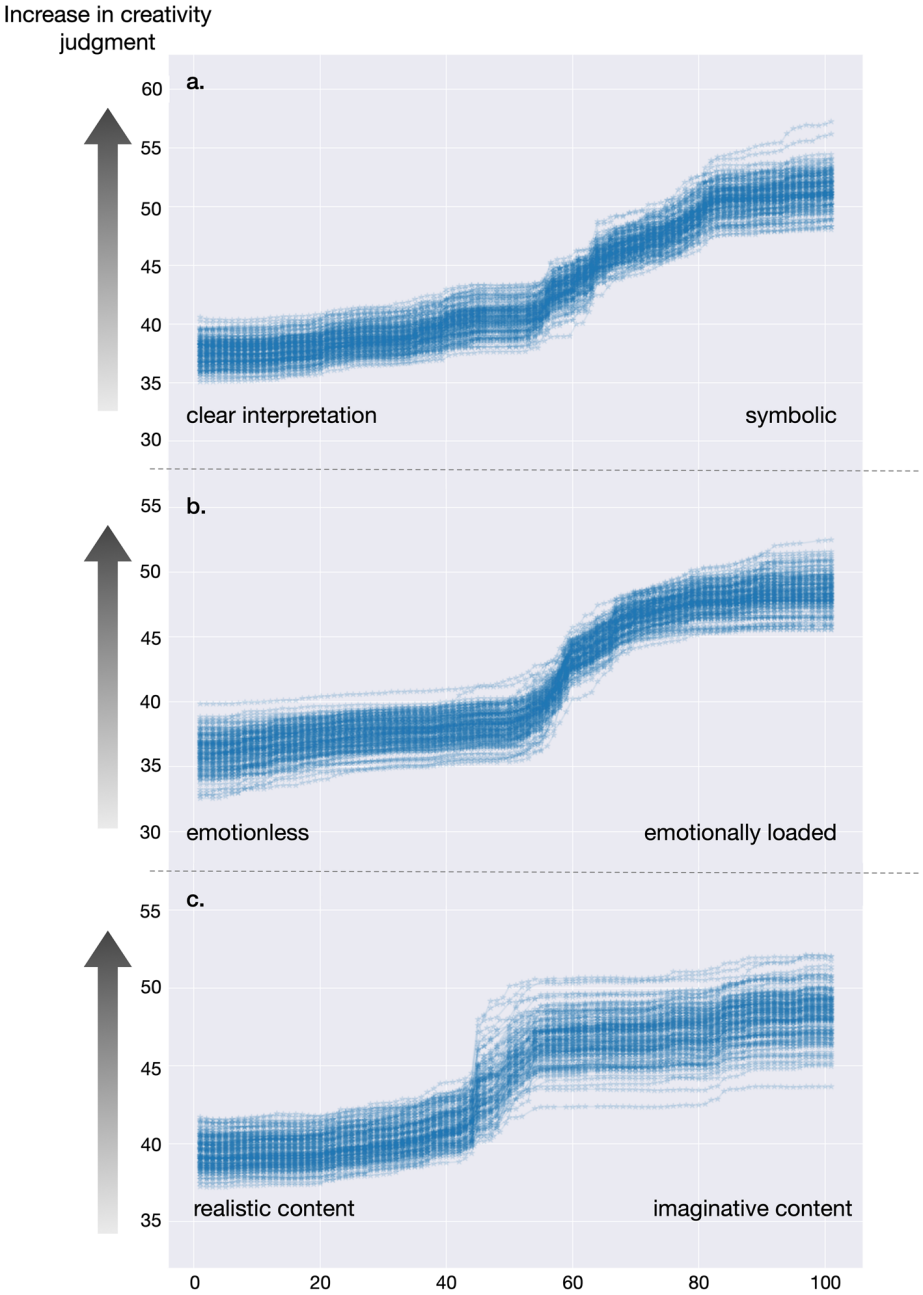
Partial dependency plots showing the association between the most important art-attribute dimensions and creativity ratings. (**a**) Association between symbolism and creativity, (**b**) association between emotionality and creativity, (**c**) association between imaginativeness and creativity.

The most important (statistically significant incl. Bonferroni correction) visual art-attributes sorted by their effect size were^63,73^: 1. *symbolisms* with the two poles distinct/clear versus symbolic (Fig. 2a) and a median effect size of 0.10, meaning that the RF prediction performance dropped from a *R*^2^ of 0.3 to 0.2 as the symbolisms values were shuffled; 2. *emotionality* with emotionless and emotionally loaded (Fig. 2b) and a median effect size of 0.07, hence, the RF prediction performance dropped from a *R*^2^ of 0.3 to 0.23; and 3. *imaginativeness* with realistic content versus imaginary, unreal, fantastic content (Fig. 2c) and an median effect size 0.05, accordingly, the RF prediction performance dropped from a *R*^2^ of 0.3 to 0.25. Effect sizes of ≥ 0.04 are usually considered practically relevant if reported in *R*^2^^73^. Less important (statistically significant excl. Bonferroni correction) were *complexity* (simple/complex), *abstraction* (representative /abstract), and *valence* (negative/ positive). The important art-attributes show a positive association with creativity ratings, thus, a higher art-attribute rating results in higher creativity judgments. Partial dependency plots show, however, that this association cannot be described via a constant factor (Fig. 2, for partial dependency plots for complexity, abstraction and valence see also Fig. S1 in Supplementary Information). Instead, sudden non-linear changes become apparent. No other art-attribute contributed significantly (see Fig. 1). Some examples of artworks rated high versus low in symbolism and imaginativeness as well as high versus low emotionality can be found in the Supplementary Information Fig. S2.

The analysis of our data uncovered an interplay among the relevant predictors, i.e., the attributes, and the target variable, creativity. Further analysis revealed, that while no significant correlation was found between the predictors and creativity, there were some observable correlations among the predictors themselves. This pattern is depicted in a correlation heatmap (see Fig. S3 Supplementary Information). Specifically, the correlation coefficients between symbolism and emotionality, and between imaginativeness and emotionality were low, with values of 0.22 and 0.16 respectively. A high correlation was found between imaginativeness and symbolism with a coefficient of 0.78, but no correlations above that. These finding still align with our initial hypothesis as the predictors were deliberately chosen to encapsulate the multifaceted and interrelated attributes of artistic creativity^3,4,64^.

The Principal Component Analysis (PCA, see Fig. S4 in Supplementary Information) showed that all but one of the components were necessary to account for more than 99% of the variance, suggesting that the original variables cannot be reduced linearly into a lower-dimensional space without a significant loss of information. These results suggests that even though predictors are correlated, they each contain unique aspects of information, instrumental in our exploration of creativity in visual art.

## Discussion

**Figure 3:**
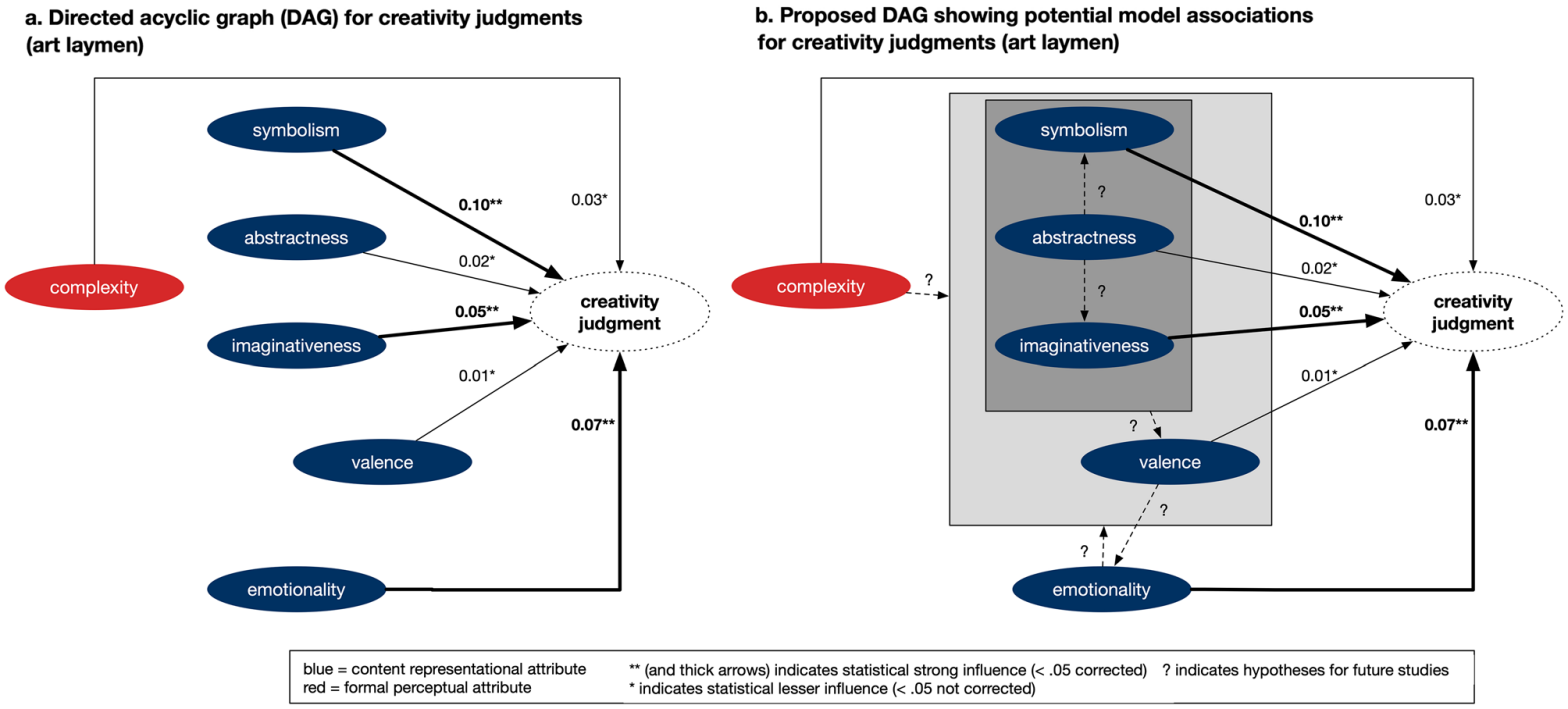
(**a**) Importance of art-attributes for predicting creativity visualized in a directed acyclic graph (DAG). Importance was assessed by a permutation procedure (i.e., the reduction in *R*^*2*^ after shuffling values of a specific art-attribute). Thickness of the arrows indicates either strong (**< 0.05 corrected) valid for attributes symbolism, emotionality, and imaginativeness; less strong influence (*< 0.05 not corrected) is valid for complexity, abstractness, and valence. (**b**) DAG model including potential additional associations and directions of influence based on the literature. Clarifying these potential associations will contribute to a future causal model. Blue circles represent content representational attributes, red circles represent formal perceptual attributes.

In our study, we examined the criteria for evaluating an artwork as creative using machine learning models. These models analyzed 17 art-attributes and revealed that artworks with higher levels of symbolism, emotionality, and imaginativeness were perceived as more creative by our sample of novice art participants. Attributes such as complexity, abstraction, and valence held less significance in determining creativity. None of the remaining art-attributes had a significant impact. Figure 3a visually presents these findings in the form of a directed acyclic graph.

The subsequent paragraphs delve into the interpretation of our findings from the perspectives of diverse research fields, including the humanities, philosophy, art history, and psychology, which have all focused on the concept of creativity. In addition to our discussion, we propose a model for future research that explores potential associations between art-attributes and creativity judgment behavior (see Fig. 3b). We also address the implications of our findings for future research, as well as the merits and limitations of our machine learning approach.

The general correlation between predictors and target were evaluated by creating a heatmap depicting all possible correlations (see Fig. S3 in Supplementary Information). This heatmap shows that there was no significant correlation between the predictors and the target variable (creativity). Our Principal Component Analysis (see Fig. S4 in Supplementary Information) further suggests that while these predictors are somewhat interrelated, they each contribute unique information to the understanding of creativity.

The most influential art-attribute was symbolism. As depicted in Fig. 2a, ratings of symbolism were associated with an increase in creativity judgments scores, particularly noticeable at a medium level of symbolism rating scores, increasing steadily even for the highest ratings. Symbolism was represented by the dimensions distinct/clear (unambiguous interpretation) versus symbolic (more space for interpretation, ambiguous). Ambiguity within symbolic presentation is an essential characteristic of art^74,75^; an attribute that has generated much discussion and research since the beginning of empirical art research about 100 years ago^49,76^. This distinction between artists presenting clear interpretation or using ambiguous more symbolic elements entails the ability of the artist to translate symbols (or on a meta-level, analogies) artistically and masterfully into a work of art. This distinction finds it roots in philosophical discourses by Heidegger^77^, Dewey^78^, and other philosophers (e.g.^79^) and is most prominent contrasting different styles. For instance, realism often showcases the artist’s creative abilities through the symbolic representation of meanings^80^, expressionism, however, seeks to convey emotional content through the creative use of provocative or perceptually dissonant symbols^41,81^, while abstract art tries to tease the viewer’s imaginative ideas^80,82^. Within all these styles, certain aspects (e.g., the specific color usage, the kinds of brushstrokes, aspects/preference of beauty, and aesthetic appeal) might lose relevance or vary depending on temporal and societal context, nonetheless, the interplay between the level of symbolism (ambiguity) and the perceived creativity appears to remain a consistent factor. This interplay is supported by studies demonstrating that great—creative—art maintains a level of enduring meaning diversity^83^, even after the ambiguity of the depicted symbolism has been resolved^74,77^. Note, the same seems true for innovative design^84^.

The second most influential art-attribute is emotionality. Figure 2b shows a similar association as with symbolism: high emotionality art-ratings are associated with higher creativity judgments ratings. This association exhibits an even sharper increase at the medium level of emotionality ratings. The role of perceived emotionality presents a complex facet of study: we had asked whether the artists managed to express emotion within the artwork. However, there might be many other attributes mediating emotionality^14,48,75,85–88^. Firstly, etymologically intertwined with emotionality is its relationship with symbolism, abstraction, and imaginativeness^85–87^. These three attributes within art representations allow individuals to be inspired by the artwork and impinge one’s personal meaning of emotional expression, drawing on personal experiences and value systems^50,89,90^. Consequently, emotional associations (experience) are probably part of this emotionality rating^62^. However, we want to note that emotionality had a very distinct impact: we found only low correlation between symbolism and emotionality (*r* = 0.22), and between imaginativeness and emotionality (*r* = 0.16).

Secondly, the quality of emotionality, might be determined by valence. Valence likely emerges from the presented content in conjunction with attributes such as symbolism, abstraction, and imaginativeness (^40^, see Fig. 3b for potential associations). However, emotionality and valence (see Fig. S3 in Supplementary Information) showed very low correlations with the other attributes in general. This leaves open questions and implications for future research seeking to deepening the understanding of the concept of emotionality as creative expression in art itself.

The third most influential predictor is associated with the human faculty of imagination (Fig. 2c). Our results indicate that artworks higher judged on this variable, thus with more imaginative, fantastical, or surreal content were judged as more creative by participants, while on the other end, more realistic artworks received lower creativity scores. This association is, again, not linear and shows a rather sudden change in influence at medium rating scores. Though seldom addressed in the context of creativity judgments in art research^4^, imagination holds a pivotal role in creativity research more broadly (referring especially to Torrance and colleagues work^6^). It is a critical component for divergent thinking^65,91^, for innovation^92^, driving new designs and developments^84^. Imagination is also a central element in various creativity scales^1,2^. Consequently, this attribute might even be crucial as a factor across different domains of creativity^1^, potentially bridging the experience of viewing art with the act of creating it. Interestingly, imaginativeness and symbolism had a comparatively high correlation coefficient of 0.78 in our study. Thus, it cannot be excluded that imaginativeness might turn out to be a common denominator between creative domains, however, in the combination with symbolism might specifically address the art sector. This might account especially for non-experts^37^. As such, the role of imagination merits further exploration in future research.

In addition to symbolism, emotionality, and imaginativeness, also the attributes complexity, abstractness, and valence predicted creativity judgments to a lesser extent, all showing a positive association with judged creativity (see Fig. S1a–c in Supplementary Information).

Complexity has been a topic in many art-research studies (e.g.^42,93,94^), and was discussed as driving attribute for preference and beauty aspects. Herein, empirical art research studies found medium levels of complexity to be most preferred and liked by especially laymen^29^. Our results regarding creativity judgments, however, show a rather steady increase in creativity judgments ratings along increasing medium levels of complexity ratings scores (see Fig. S1a in Supplementary Information). Interestingly, complexity also contributes to the aspects of ambiguity^70,74^. Our correlation heatmap shows a low-level association with symbolism (*r* = 0.38) and imaginativeness (*r* = 0.36). This might be because as complexity increases, so do visual perceptual factors, which can lead to a multilayered ambiguous impression and thereby also increasing the impression of symbolism, imaginativeness, and abstractness and vice versa^95^. However, as some research also claimed that a very high level of complexity might be perceived as uncomfortable and thus negative^43,44^, we did not find a correlation between complexity and valence. As our main results showed that complexity is not as important as other factors, one might conclude that this formal-perceptual feature is partially ingrained in content-representational features and thereby mediating the process (see Fig. 3b). This idea needs to be tested in in-depth research; however, our research might deliver a new basis to solve the many controversial hypotheses and findings regarding the effects of complexity on art judgments^93,96^, including the aspect of valence. Note, that the average level of complexity in relation to higher creativity, might differ between different art expertise levels^97^, which suggest testing the model for different levels of expertise.

A similar argumentation might hold for abstractness. Abstractness is a content stylistic aspect that is qualitatively deeply connected with symbolism and imaginativeness^8,14^. And, indeed, in our study there were high correlations between abstractness and symbolism (*r* = 0.72), and imaginativeness (*r* = 0.78) but not emotionality (*r* = 0.22). Interestingly, as abstract art has been discussed as a universally readable attribute of art^82^, abstractness is also often not liked by non-experts; hence, abstractness offers interesting research questions for expertise-related, but also cross-cultural comparisons^37,54,55^.

Finally, valence, as influential predictor, with a range from negative to positive valence, is a standard variable employed in art research^14^. Art that evokes positive or negative valence, can be appealing and hereby influence the perception of emotionality^98^. Our results focusing on creativity, nonetheless, showed a positive association between valence scores with creativity judgments, hence, more positive valence scores were associated with higher creativity at a nearly constant rate. This result might be explained by the low level of art expertise of our participants, favoring mainly positive art^75^. Art experts compared to laymen, however, appreciate both positive and negative art, thus, judging art more independently of the level of valence^37^. In addition to investigating the model in different cohorts of expertise, an interesting future viewpoint should also consider that all mentioned predictors (except for emotionality) might mediate valence and the judged creativity (Fig. 3b)^29,64^. Our results furthermore indicate that for creativity, emotionality is a stronger predictor than whether the artwork was negative or positive in valence^98^. Hence emotionality could be a much more central factor, where valence might be less important for the evaluation of creativity. However, again, one can speculate that this depends on the current context and person-related factors.

As mentioned already several times throughout the paper, in our study with non-art expert participants, it is crucial to consider the potential differences in rating behavior between art experts and non-experts^37,54,97^. Prior literature has highlighted substantial variations in art judgments between these two groups. Non-experts tend to place greater emphasis on the content of artworks, as reflected in our findings where content-driven attributes, such as symbolism, emotionality, and imaginativeness, played significant roles in predicting creativity judgments^55,100^. However, it is plausible that an analysis of expert judges’ ratings using the same art-attributes of our study could yield a different pattern of results. Considering past literature, we would assume that art experts may use more formal-perceptual attributes to evaluate an artwork, such as specific color usage or technical skill requirements like brushstroke or visualization of depths^37,101,102^. As mentioned before, also the interplay of complexity and valence direction could differ between art novices and art experts, as they engage an artwork with different knowledge seeing the skill in depicting, for example, negative art or less emotional expressive art. In addition, we would expect that experts will use more art-attributes for their evaluation in general.

As for our main attributes of symbolism, emotionality, and imaginativeness, it is uncertain if they would retain the same significance in an art experts’ group, although they probably represent general markers of creativity in visual art that are independent of expertise. Further research is needed to test these assumptions and explore the systematic differences in attribute significance between art experts and non-experts.

Although our machine learning-based analysis contained many attributes that could have contributed to creativity, only few attributes explained most of the variance. Additionally, five of six significant attributes—including all three main predictors—are attributes that belong to the content-representational category (see Table 1). We would like to emphasize this since so far as machine learning has only been applied with objective attributes gained from image analysis, which rather correspond to formal-perceptive attributes^18–21^. According to our results, however, humans appear to judge artwork’s creativity according to the level of content and aspects of meaning.

Our approach intends to show that art-novice behavior, using the evaluation of creativity of visual art, is inherently complex and non-linear due to the multitude of factors influencing each decision, many of which are interrelated and affect each other in a non-sequential manner. Individuals’ perceptions of art can be influenced by a range of factors including personal experiences, cultural background, emotional state, and inherent biases, all of which can change over time and in different contexts. This dynamism and its non-linearity make it challenging to accurately predict using traditional linear models. Machine learning models, on the other hand, excels in handling such complexities. Its ability to model intricate patterns and interrelationships in high-dimensional space allows for a more nuanced understanding and prediction of non-linear human behavior, making it a powerful tool in art research.

The observed stepwise shape in the graphs, especially for emotionality and imaginativeness could indicate a threshold phenomenon, a concept reminiscent of findings in psychophysics where perceptual changes do not scale linearly with stimulus variations. At these transition points, further increases in these attributes might be less efficient. This suggests that artworks may be deemed equally creative beyond a certain attribute intensity, potentially indicative of an aesthetic threshold. Previous research in the art domain has indeed suggested the existence of non-linear relationships^3,4,29,44,103^, although a comprehensive investigation into this aspect remains under-explored, especially in the field of creativity and psychological empirical art theories. Our machine learning approach holds promise in delving deeper into the concept of an aesthetic threshold. It remains possible that these patterns may reflect inherent aspects of human aesthetic perception, and if so, our method could potentially illuminate its characteristics and implications, thereby enriching the understanding of aesthetic evaluations in art and other creative traditions.

Regarding the methods employed, our approach was a combination of RF ensemble regression^39^ with techniques from the field of interpretable machine learning to gain insights into the associations learned by the model^46^. With the prediction of creativity judgements ratings as a target of art-attributes, we introduce a comprehensive method and a newly established initial model for art judgment analysis. The introduction of machine learning into art research is a consequent development of various methods to identify predictors of aesthetic appreciation and art evaluations, beginning with pure correlation or regression analyses^27–29^ and more recent approaches using machine learning with objective values^8,19–21^.

However, machine learning also faces general limitations of data-driven modelling method. These comprise aspects such as no causal associations, unexplained variance in data (in our study 70% on the variance stays unexplained), unclear transferability of associations learned by models to human behavior, model selection bias (other algorithms are available), data sample bias (limited variability, e.g., age, culture, etc.), variables selection bias (including other confounding variables may lead to different results), and stimulus material selection bias. Nevertheless, interpretable machine learning overcomes some limitations of traditional data modelling. That is, interpretable machine learning offers flexible, complex, yet robust, credible, and assessable models^39^. These models are not limited or biased by problematic assumptions on pre-determined variable interactions, by variable scales, or by model oversimplification^39^. Such assumptions and simplifications are problematic since they can lead to irrelevant or questionable scientific theories and conclusions (e.g., on causality^39^). This approach delivers the first steps towards establishing the basis for new hypothesis-driven research^39^, where quantitative statistical-learning models are the basis for future research on causal relationships.

Furthermore, by working with those methods that use less assumptions about the data, we were able to identify a complex interplay of variables beyond associations that can be described by a constant factor^39,46^. This is relevant for the social psychological field^30,34^ looking at judgment pattern behavior and penetrating the existing approaches of art research (see for review^8,14^): namely, that art judgments are made along complex patterns including sudden changes and that only a limited number of variables learn a quantitative prediction model.

While our study aimed to introduce a comprehensive approach by considering a diverse range of attributes, it needs to be acknowledged that our list of attributes is not exhaustive. There may be additional relevant factors that were not included in our analysis. Furthermore, it is crucial to note that our focus was primarily on Western art, which may limit the generalizability of our findings to other artistic traditions and cultural contexts. Additionally, our participant sample consisted solely of laymen and psychology students, which may restrict the representativeness of the results. Therefore, further research is warranted to address these limitations as implications for future research: expanding the attribute set, encompassing a broader range of artistic traditions and cultures, and incorporating diverse participant samples (e.g., art experts).

Furthermore, a potential constraint concerns the distribution of our data. The characteristics of our data distributions might have influenced the form of the predictors’ impact, leading to a step function-like shape (see Fig. S5 in Supplementary Information). This distribution pattern could have implications for the interpretation of our results and should be taken into consideration. In future studies, it would be beneficial to further explore the influence of data distribution, possibly by applying different statistical methods or transformations to ascertain the robustness of our findings.

The present study underlines the potential of machine learning in art research, and more specifically, in the study of creativity judgements^54^. In doing so, we fundamentally and conceptually intervene in the question of which attribute dimensions contribute to the judgment of art as creative. We also intervene in social-cultural and communication aspects: the attributes (see Table 1) used in our study are all attributes that are also used as interpersonal linguistic tool to justify judgments^5,89^, in the field of fine art and other creative areas. Judgments where we humans seem to trust ourselves to judge how creative a product or work of art is^3,30,31^.

Finally, as described in the Introduction, creativity is relevant in many areas^1,2^. Therefore, our study might be a promising approach for the future of creativity research in general^1–3^. It could be applied to studying traits, states, skills, which in turn is tied to psychological phenomena and outstanding personalities such as geniuses^32,104^; also, creative design and product development (non-virtual and virtual) outside the visual art domain could employ consumer-based judgment analysis. Again, interpretable machine learning^46^ can reveal a more comprehensive picture of what creativity entails in individual domains; through comparison it can uncover how specific attributes are generalizable across domains in the future and how these might be universal between individuals, traditions, and cultures^105^. Indeed, as Immanuel Kant proposed, visual art is subject to the subjective perception of each viewer while simultaneously encapsulating universal aspects of human experience.

### Supplementary Information

Supplementary Information.

## Data Availability

The datasets generated and/or analysed during the current study are available in the Figshare repository, 10.6084/m9.figshare.19097099.
