# Supplementary material for: Machine learning revealed symbolism, emotionality, and imaginativeness as primary predictors of creativity evaluations of western art paintings
Source: Sci Rep. 2023 Aug 10;13:12966. doi: 10.1038/s41598-023-39865-1 (PMC10415252; doi:10.1038/s41598-023-39865-1)
Supplement: Supplementary file 1 — Supplementary Information. [file 41598_2023_39865_MOESM1_ESM.docx]

**Supplementary Information for**

**Machine Learning Revealed Symbolism, Emotionality, and Imaginativeness as Main Predictors of Creativity Evaluations of Western Art Paintings**

Blanca T.M. Spee^1,2^**^,^**^*^, Jan Mikuni^3^, Helmut Leder^1,3^, Frank Scharnowski^3,4^, Matthew Pelowski^1,3^, David Steyrl^3,4^

^1^Vienna Cognitive Science Hub, University of Vienna, 1010 Vienna, Austria

^2^Department of Neurology, Radboud University Medical Center, Nijmegen, the Netherlands

^3^Department of Cognition, Emotion, and Methods in Psychology, Faculty of Psychology, University of Vienna, 1010 Vienna, Austria

^4^Department of Psychiatry, Psychotherapy and Psychosomatics, Psychiatric University Hospital, University of Zurich, 8008 Zurich, Switzerland

*Corresponding author: Blanca T.M. Spee, University of Vienna, Faculty of Psychology, Liebiggasse 5, A-1010 Vienna, Austria.

**Email:** blanca.spee@univie.ac.at

**Supplementary Information Text**

**Stimulus selection and pre-ratings.**

We selected the stimulus by using the provided judgment ratings (liking, valence, arousal, complexity, familiarity) of the Vienna Art Picture System (VAPS), a dataset of 999 fine art paintings and subjective ratings for art and aesthetics research. The artwork image catalogue along with the rating are stored at the main institution and is currently not available to the public due to copyrights issues and protection of the artists. The picture numbers in the current study are corresponding to the ones which are in the catalogue. To ensure that the sub-set of images for the current study are balanced, ensuring variety of, e.g., liking ratings in the selected pictures, we applied Wilk-Shapiro tests for all depicted motif/style combined, and separately for both depicted motif and style (for Results see Table S3 in Supplement Materials) to ensure the normality in the given rating scores. Overall, nearly all categorizations had a normal distribution. However, there are several ratings which are not normally distributed in our stimulus set. First, the art judgment familiarity was not normally distributed in all categories. Presumably, this is because the VAPS contains extremely famous and not famous artworks. Further, the VAPS-raters were all art novices. Therefore, a normal distribution was not possible with the present image-set. Second, the VAPS-raters perceived representative art significantly as more positive in valence and arousing. These results are probably also justifiable, since the most famous artworks most likely known by the VAPS sample (young students, art laymen) were representative artworks, which could have led to higher positive valence, and affective resonance (arousal) due to re-exposure effect. However, as the applied machine learning based analyses approach does not require normally distributed data and because normal distributions were obtained in all other categories, we had no doubt that this is not a limitation for the current study.

**Supplementary Information Figures**


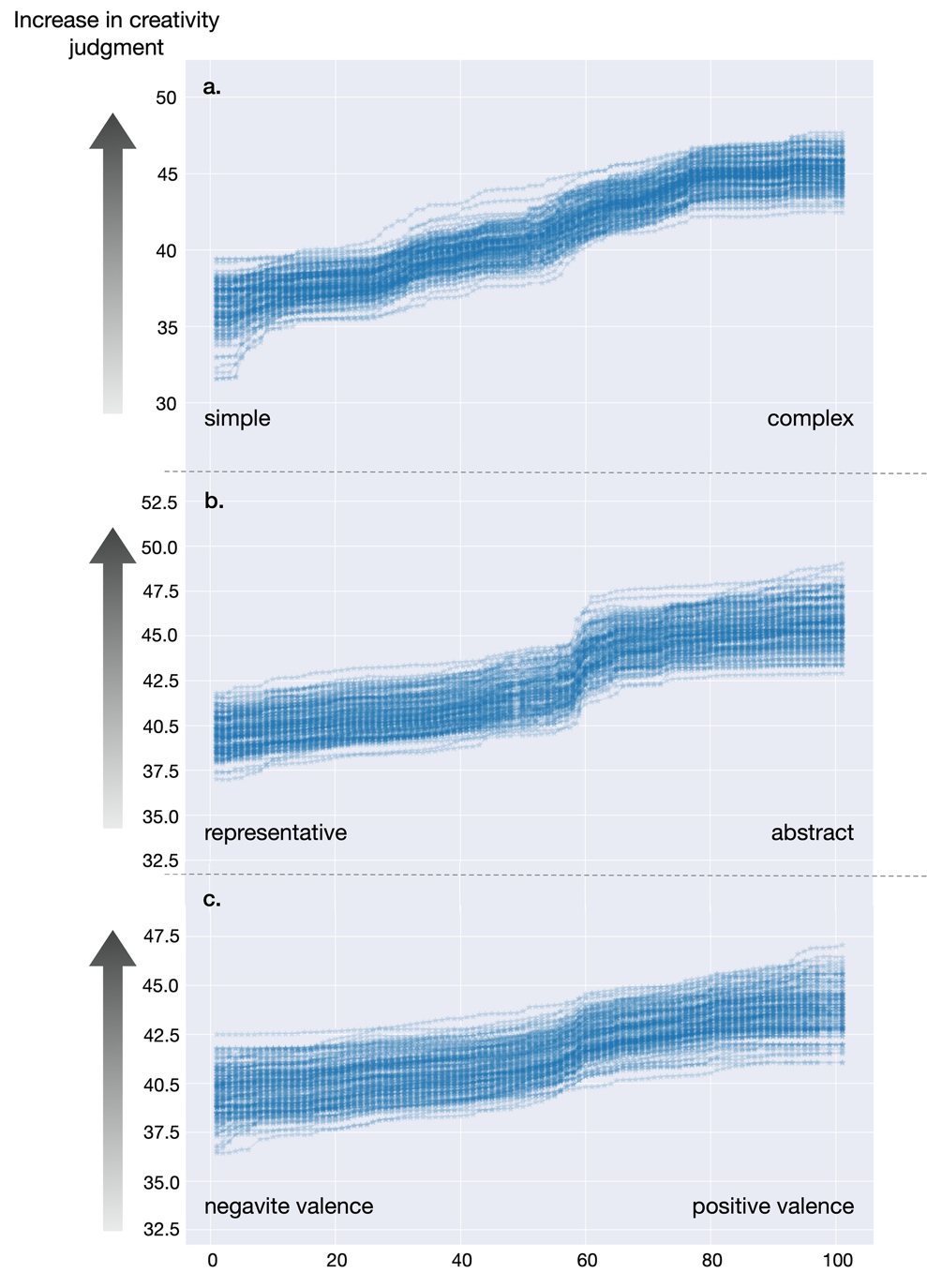


**Figure S1.** Partial dependency plots showing the associations between the other important art-attribute dimensions and the dependent variable creativity. a. association between complexity and creativity, b. association between abstraction and creativity, c. association between valence and creativity.


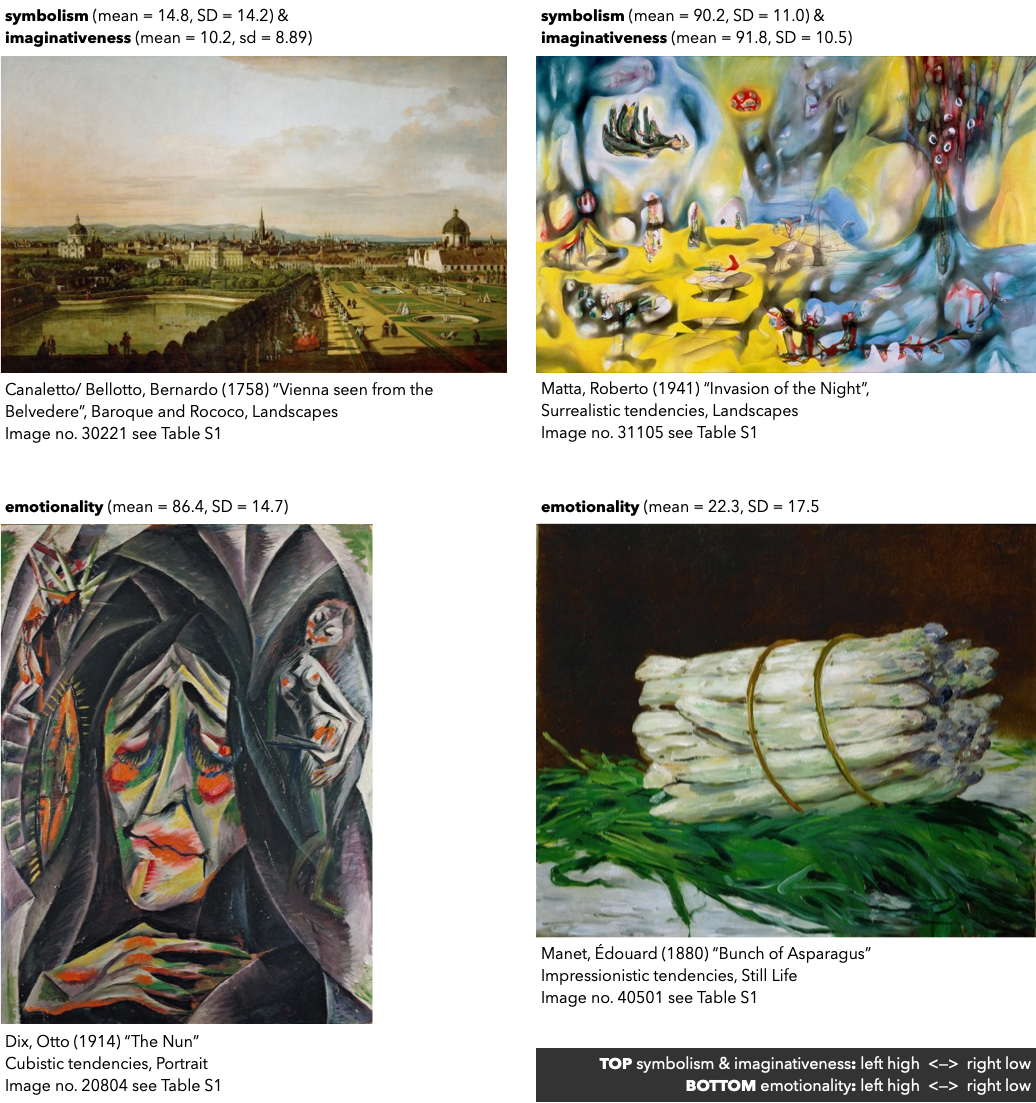


**Figure S2.** Some examples of artworks rated high versus low in symbolism and imaginativeness as well as high versus low emotionality. Copyright information: Shown works are in the public domain in its country of origin and other countries and areas where the copyright term is the author’s life plus 70 years or fewer.


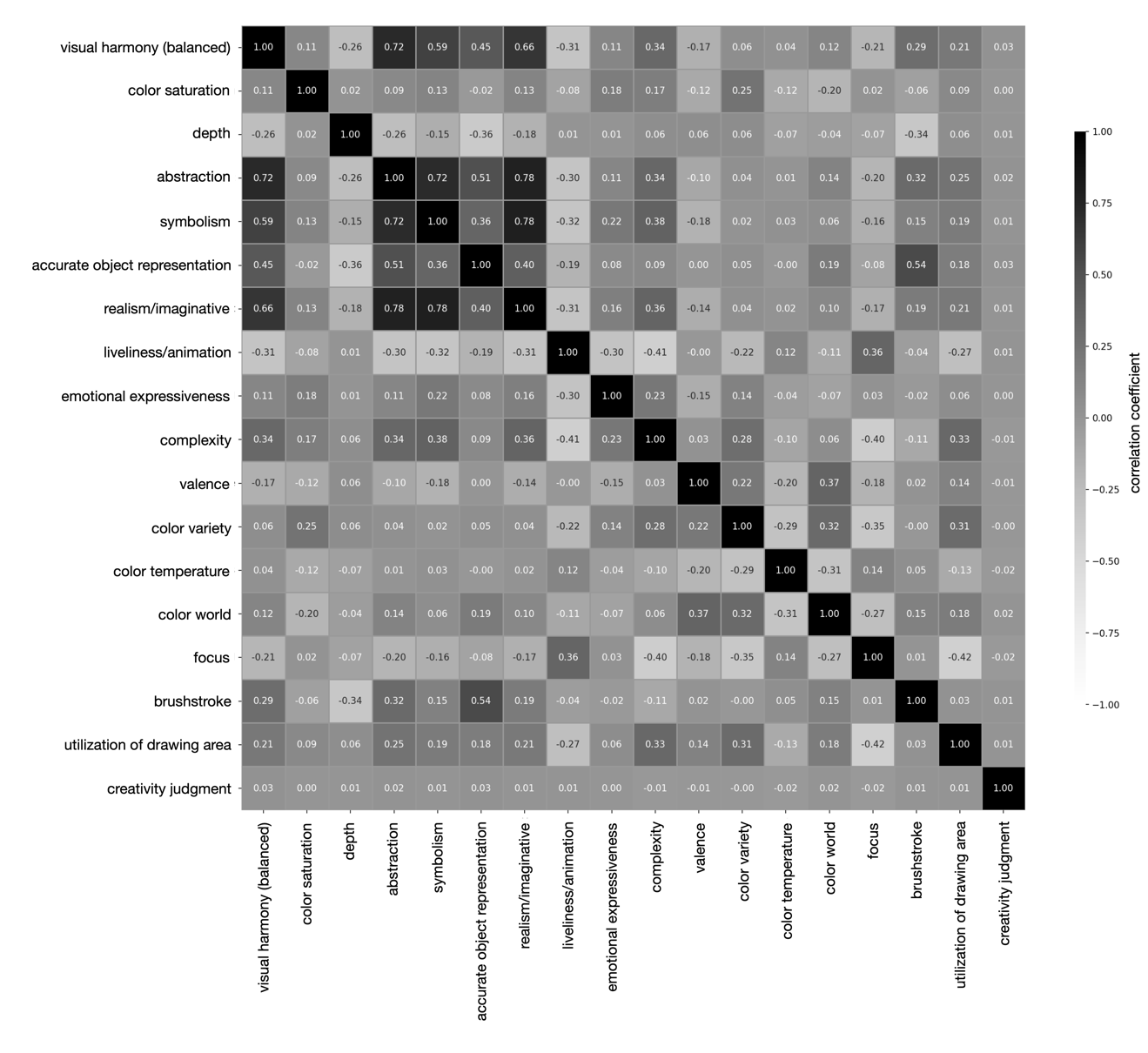


**Figure S3.** Exhaustive heatmap of all correlations between attributes and creativity judgment. Statistical significance was not assessed. This heatmap represents the correlation coefficients among predictors and creativity. While correlations between predictors are observed, they do not exhibit a strong correlation with creativity. Despite some predictors showing interdependence (historically expected), our method accommodates this, as the coefficients do not reach 1, ensuring each predictor still carries unique information.

**
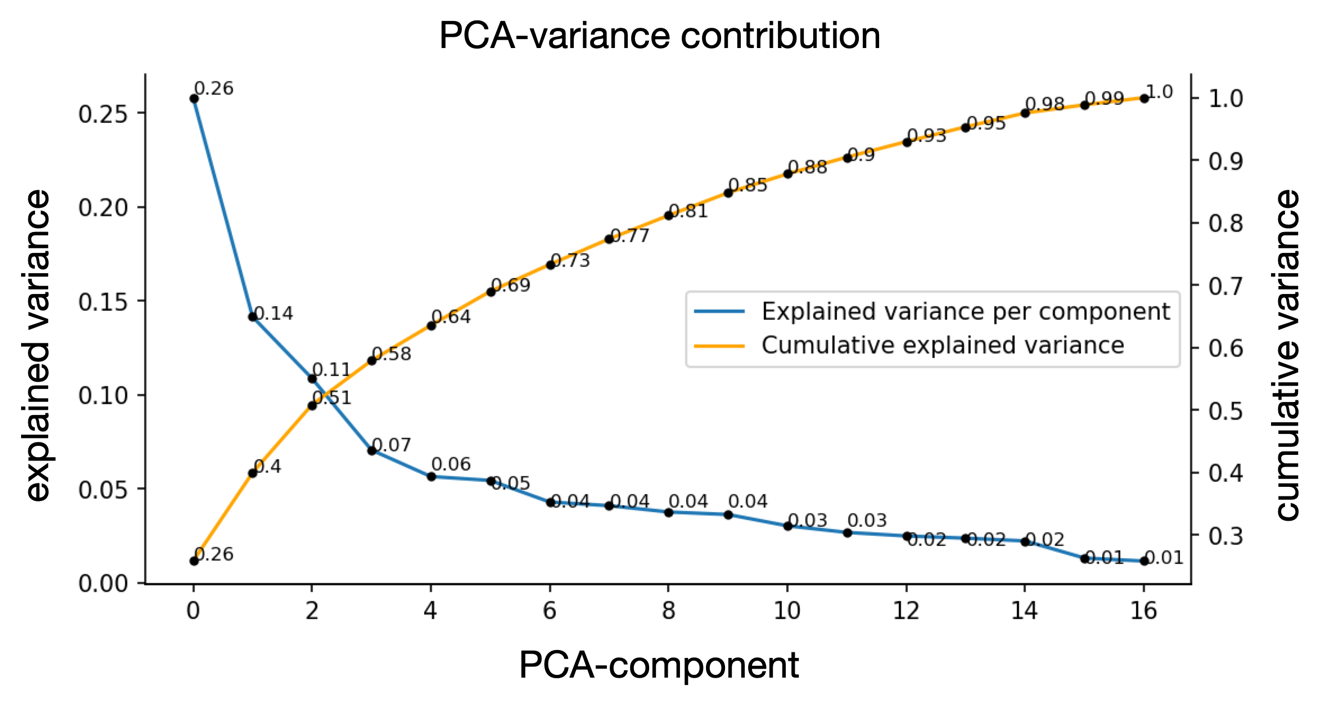
**

**Figure S4.** Principal component analysis. This figure depicts the results of the PCA, demonstrating the percentage of the total variance explained by each principal component; all but one of the components were necessary to account for over 99% of the total variance in the data set; this suggests that reducing the data to a lower-dimensional space would result in significant information loss.


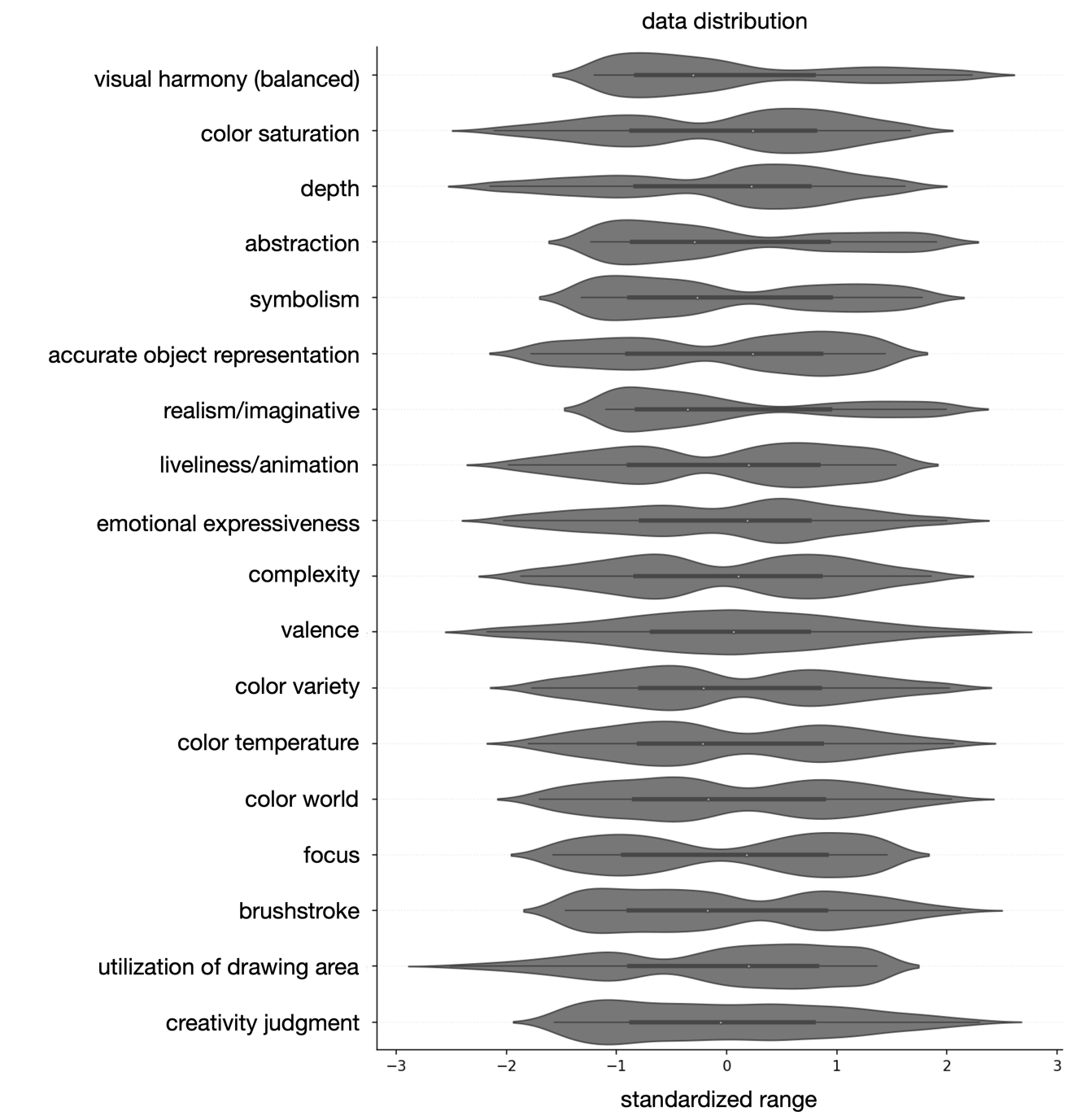


**Figure S5.** Distribution of attributes.

**Supplementary Information Tables**

**Table S1.** Items of art-attributes along their semantic differential dimension poles (independent variables used in machine learning analysis). German version.

|  | **Anweisung** | *Bitte bewerten Sie das Kunstwerk anhand der unterschiedlichen Attribute:* | |
| --- | --- | --- | --- |
| **Attribute** | **Items** | **Negativer Pol (Minimum)** | **Positiver Pol (Maximum)** |
| **i. Formal-perzeptive Attribute** | a. Visuelle Harmonie (Balance) | visuelle Harmonie, proportional | eigenartig, seltsame Formen |
|  | b. Tiefenwahrnehmung | zwei-dimensional | drei-dimensional |
|  | c. Komplexität | einfach | komplex |
|  | d. Farbsättigung | sanft, pastell | intensive, kräftig |
|  | e. Farbvielfalt | wenige Farben | Farbvielfalt |
|  | f. Farbtemperatur | warme Farben | kalte Farben |
|  | g. Farbwelt | dunkle Farbwelt | helle Farbwelt |
|  | h. Pinselführung | feine Pinselführung | grobe Pinselführung |
|  | i. Ausnützung der Zeichenfläche | wenig Ausnützung der Malfläche | sämtliche Ausnützung der Malfläche |
| **ii. Inhaltlich-repräsentative Attribute** | j. Abstraktion | repräsentativ | abstrakt |
|  | k. Imagination | realistischer/s Inhalt/ Thema | imaginär, unwirklich, fantastisch |
|  | l. Symbolismus (Ambiguität) | eindeutig (klare Interpretation der Darstellung) | symbolisch (mehr Interpretationsfreiraum) |
|  | m. Akkurate Objektdarstellung | fotorealistisch | malerisch |
|  | n. Lebendigkeit, Animation | dynamisch | still |
|  | o. Emotionalität | emotionslos | emotional aufgeladen |
|  | p. Valenz | negative Stimmung | positive Stimmung |
|  | q. Fokussierung | viel Kontext/Umgebung  im Bild | fokussierter Inhalt |

Table S2. Full list of stimulus-set including name of artist, title, style, and depicted motif. ID represents the VAPS-identification code.

| **ID** | **Name of Artist** | **Title** | **Style** | **Category** |
| --- | --- | --- | --- | --- |
| 20202 | Gentileschi, Orazio | Portrait of a Young Woman as a Sibyl | Baroque and Rococo | Portrait |
| 20209 | Rubens,  Peter Paul | Portrait of a Woman, Probably Susanna Lunden | Baroque and Rococo | Portrait |
| 20211 | Vermeer, Jan | Das Mädchen mit dem Perlenohrgehänge | Baroque and Rococo | Portrait |
| 20226 | Nattier,  Jean-Marc | Elizabeth, Countess of Warwick | Baroque and Rococo | Portrait |
| 20228 | Gainsborough, Thomas | Portrait of Mrs. Gainsborough | Baroque and Rococo | Portrait |
| 20233 | Reynolds, Joshua | Self Portrait | Baroque and Rococo | Portrait |
| 20501 | Degas, Edgar | Duchess of Montejasi | Impressionistic  tendencies | Portrait |
| 20507 | Renoir,  Pierre-Auguste | Portrait of Madame Alphonse Daudet | Impressionistic  tendencies | Portrait |
| 20509 | Renoir,  Pierre-Auguste | Hyacinthe-Eugène Meunier (1841-1906), Called Eugène Murer | Impressionistic  tendencies | Portrait |
| 20608 | Cezanne, Paul | Portrait of Madame Cezanne | Postimpressionistic  tendencies | Portrait |
| 20615 | Picasso, Pablo | Femme la Chemise | Postimpressionistic  tendencies | Portrait |
| 20616 | Gogh van, Vincent | Italian Woman (Agostina Segatori) | Postimpressionistic  tendencies | Portrait |
| 20804 | Dix, Otto | The Nun | Cubistic  tendencies | Portrait |
| 20806 | Metzinger, Jean | Le Goûter, Tea Time | Cubistic  tendencies | Portrait |
| 20810 | Feininger, Lyonel | Selbstbildnis | Cubistic  tendencies | Portrait |
| 21105 | Magritte, Rene | Man in a Bowler Hat (Man mit Melone) | Cubistic  tendencies | Portrait |
| 21106 | Magritte, Rene | Clairvoyance | Surrealistic  tendencies | Portrait |
| 21107 | Magritte, Rene | Rape (La Violazione) | Surrealistic  tendencies | Portrait |
| 30210 | Everdingen van, Allaert | Swedish Landscape | Baroque and Rococo | Landscape |
| 30211 | Goyen van, Jan | Landscape with Dunes | Baroque and Rococo | Landscape |
| 30218 | Vermeer, Jan | View of Delft, Netherlands, after the Fire | Baroque and Rococo | Landscape |
| 30220 | Canaletto/Bellotto, Bernardo | View of Dresden from the Left Bank of the Elbe, below the Fortifications | Baroque and Rococo | Landscape |
| 30221 | Canaletto/Bellotto, Bernardo | Vienna seen from the Belvedere | Baroque and Rococo | Landscape |
| 30225 | Guardi, Francesco | The Feast of Maundy Thursday in Venice | Baroque and Rococo | Landscape |
| 30505 | Monet, Claude | La Gare Saint-Lazare | Impressionistic  tendencies | Landscape |
| 30506 | Monet, Claude | Haystacks, End of Summer, Giverny | Impressionistic  tendencies | Landscape |
| 30511 | Sisley, Alfred | Pradera | Impressionistic  tendencies | Landscape |
| 30611 | Cezanne, Paul | The Lac d'Annecy | Postimpressionistic  tendencies | Landscape |
| 30613 | Cezanne, Paul | Mont Sainte-Victoire | Postimpressionistic  tendencies | Landscape |
| 30615 | Gogh van, Vincent | Cafe Terrace at Night  (Place du Forum in Arles) | Postimpressionistic  tendencies | Landscape |
| 30804 | Delaunay, Robert | Eiffel Tower with Trees | Cubistic  tendencies | Landscape |
| 30807 | Rivera, Diego | Trees and Walls in Toledo | Cubistic  tendencies | Landscape |
| 30808 | Marc, Franz | Kleine Komposition II  (Haus mit Bäumen) | Cubistic  tendencies | Landscape |
| 31103 | Magritte, Rene | Blood with Tell  (La voix du sang) | Cubistic  tendencies | Landscape |
| 31105 | Matta, Roberto | Invasion of the Night | Surrealism Cubistic  tendencies | Landscape |
| 31106 | Matta, Roberto | The Onyx of Electra | Cubistic  tendencies | Landscape |
| 40209 | Aertsen, Pieter | Butcher Shop | Baroque and Rococo | Still life |
| 40220 | Hoogstraten van, Samuel | Slippers, Dutch Interior | Baroque and Rococo | Still life |
| 40224 | Snyders, Frans | Three Monkeys with Fruit | Baroque and Rococo | Still life |
| 40229 | Chardin, Jean Siméon | Dead Hare with Powder Flask and Game-bag | Baroque and Rococo | Still life |
| 40230 | Chardin, Jean Siméon | Musical Instruments and Parrot | Baroque and Rococo | Still life |
| 40234 | Vallayer-Coster, Anne | Bouquet of Flowers in a Terracotta Vase, with Peaches and Grapes | Baroque and Rococo | Still life |
| 40501 | Manet, Édouard | Bunch of Asparagus | Impressionistic  tendencies | Still life |
| 40502 | Monet, Claude | Apples and Grapes | Impressionistic  tendencies | Still life |
| 41503 | Monet, Claude | Bouquet of Sunflowers | Impressionistic  tendencies | Still life |
| 41610 | Gauguin, Paul | The Ham | Postimpressionistic  tendencies | Still life |
| 41612 | Gogh van, Vincent | Irises | Postimpressionistic  tendencies | Still life |
| 41616 | Gogh van, Vincent | Head of a Skeleton with a burning Cigarette | Postimpressionistic  tendencies | Still life |
| 40809 | Braque, Georges | Composition with Ace of Clubs | Cubistic  tendencies | Still life |
| 40810 | Braque, Georges | Still Life with Grapes and Clarinet | Cubistic  tendencies | Still life |
| 40814 | Gris, Juan | Guitar and Glasses | Cubistic  tendencies | Still life |
| 41106 | Magritte, Rene | Personal Values (Les valeurs personnelles) | Cubistic  tendencies | Still life |
| 41109 | O'Keeffe, Georgia | Head with Broken Pot | Cubistic  tendencies | Still life |
| 41110 | Ernst, Max | Ödipus Rex | Cubistic  tendencies | Still life |

**Table S3.** The results of Wilk-Shapiro test for checking the data normality of each rating scale per motif/style.

|  | ***W*** | ***p*-value** |
| --- | --- | --- |
| **All depicted motif/style combined** | | |
| Liking | 0.98 | .52 |
| Valence | 0.98 | .54 |
| Arousal | 0.95 | .06 |
| Complexity | 0.95 | .054 |
| Familiarity | 0.74 | **< .001** |
| **Depicted motif-wise (portrait)** | | |
| Liking | 0.96 | .79 |
| Valence | 0.95 | .57 |
| Arousal | 0.93 | .20 |
| Complexity | 0.95 | .55 |
| Familiarity | 0.70 | **< .001** |
| **Depicted motif-wise (landscape)** | | |
| Liking | 0.95 | .51 |
| Valence | 0.95 | .44 |
| Arousal | 0.94 | .32 |
| Complexity | 0.92 | .18 |
| Familiarity | 0.63 | **< .001** |
| **Depicted motif-wise (still-life)** | | |
| Liking | 0.95 | .48 |
| Valence | 0.95 | .44 |
| Arousal | 0.94 | .40 |
| Complexity | 0.96 | .74 |
| Familiarity | 0.81 | **< .001** |
| **Style-wise (representative)** | | |
| Liking | 0.92 | .19 |
| Valence | 0.87 | **.02** |
| Arousal | 0.78 | **< .001** |
| Complexity | 0.93 | .20 |
| Familiarity | 0.58 | **< .001** |
